# Association between 3D Printing-Assisted Pelvic or Acetabular Fracture Surgery and the Length of Hospital Stay in Nongeriatric Male Adults

**DOI:** 10.3390/jpm12040573

**Published:** 2022-04-03

**Authors:** Chun-Chi Hung, Pei-Hung Shen, Jia-Lin Wu, Yung-Wen Cheng, Wei-Liang Chen, Shih-Han Lee, Tsu-Te Yeh

**Affiliations:** 1 Department of Orthopaedic Surgery, Tri-Service General Hospital and School of Medicine, National Defense Medical Center, No. 325, Sec. 2, Chenggong Rd. Neihu Dist., Taipei City 11490, Taiwan; raffi10110126@gmail.com (C.-C.H.); sph1468@yahoo.com.tw (P.-H.S.); 2 Division of Traumatology, Department of Surgery, Tri-Service General Hospital and School of Medicine, National Defense Medical Center, No. 325, Sec. 2, Chenggong Rd. Neihu Dist., Taipei City 11490, Taiwan; 3 Department of Orthopedics, School of Medicine, College of Medicine, Taipei Medical University, Taipei 11031, Taiwan; wu.jialin@tmu.edu.tw (J.-L.W.); kozuetora@gmail.com (S.-H.L.); 4 Department of Orthopedics, Taipei Medical University Hospital, Taipei 11031, Taiwan; 5 Orthopedics Research Center, Taipei Medical University Hospital, Taipei 11031, Taiwan; 6 Centers for Regional Anesthesia and Pain Medicine, Wan Fang Hospital, Taipei Medical University, Taipei 11600, Taiwan; 7 Division of Family Medicine, Department of Family and Community Medicine, Tri-Service General Hospital and School of Medicine, National Defense Medical Center, No. 325, Sec. 2, Chenggong Rd. Neihu Dist., Taipei City 11490, Taiwan; cyw0451@gmail.com (Y.-W.C.); weiliang0508@gmail.com (W.-L.C.); 8 Division of Geriatric Medicine, Department of Family and Community Medicine, Tri-Service General Hospital and School of Medicine, National Defense Medical Center, No. 325, Sec. 2, Chenggong Rd. Neihu Dist., Taipei City 11490, Taiwan; 9 Department of Biochemistry, National Defense Medical Center, No. 161, Sec. 6, Minquan E. Rd. Neihu Dist., Taipei City 11490, Taiwan; 10 Medical 3D Printing Center, Tri-Service General Hospital and National Defense Medical Center, No. 325, Sec. 2, Chenggong Rd. Neihu Dist., Taipei City 11490, Taiwan

**Keywords:** 3D-printing assisted surgery, trauma, injury severity score, ISS, pelvis fracture, acetabulum fracture, length of hospital stay

## Abstract

Pelvic and acetabular fractures are challenging for orthopedic surgeons, but 3D printing has many benefits in treating these fractures and has been applied worldwide. This study aimed to determine whether 3D printing can shorten the length of hospital stay (LHS) in nongeriatric male adult patients with these fractures. This is a single-center retrospective study of 167 nongeriatric male adult participants from August 2009 to December 2021. Participants were divided into two groups based on whether they received 3D printing assistance. Subgroup analyses were performed. Pearson’s correlation and multivariable linear regression models were used to analyze the LHS and the parameters. Results showed that 3D printing-assisted surgery did not affect LHS in the analyzed patients. The LHS was positively correlated with the Injury Severity Score (ISS). Initial hemoglobin levels were negatively associated with LHS in patients aged 18–40 and non-major trauma (ISS < 16) patients. In 40–60-year-old and non-major trauma patients, the duration from fracture to admission was significantly associated with LHS. This study indicates that 3D-assisted technology for pelvic or acetabular fracture surgery for nongeriatric male adults does not influence the LHS. More importantly, the initial evaluation of patients in the hospital was the main predictor of the LHS.

## 1. Introduction

Fractures of the adult pelvis and acetabulum are generally either fragility fractures resulting from low-energy trauma, such as falls in elderly patients, or fractures caused by high-energy trauma that result in significant morbidity and mortality. High-energy pelvic or acetabular fractures result most commonly from automobile accidents, falls from height, motorcycle accidents, automobile–pedestrian encounters, and industrial crush injuries [1,2]. The potential complications of high-energy pelvic and acetabulum fractures include injuries to the main vessels and nerves of the pelvis and therefore the major viscera, such as the liver, spleen, and kidney. Degloving injuries to the encompassing soft tissues, both open and closed, may also accompany these fractures and complicate their treatment [3]. Reported mortality from severe pelvic and acetabulum fracture ranges from 10% to as high as 50% in some earlier series of open pelvic fractures. Risk factors for increased mortality include the patient’s age and Injury Severity Score (ISS), associated head or visceral injury, blood loss, hypotension, coagulopathy, and unstable or open pelvic fractures [4,5,6].

The interdisciplinary research area of three-dimensional (3D) printing in medicine and surgery has developed rapidly over the past two decades [7,8]. Now, 3D printing is one of the most effective and important technologies in medical applications. It provides doctors and medical service members better anatomy comprehension through 3D printing models. The use of 3D printing models for operative planning has been implemented mainly to determine the acceptable method of fracture reduction. In some cases, surgeons are able to use sterilized models intraoperatively, and bio-acceptable material has been designed as implants. In severe cases where complex fracture occurs, 3D modeling allows surgeons to preoperatively mold metal plates to match the configuration of the fracture [8,9,10,11,12].

High-energy pelvic or acetabular fractures are often managed operatively, with the treatment method determined by the stability of fracture sites remaining after the injury [13]. The typical goal for the care of an adult patient with a pelvic ring or acetabulum injury is union during a well-aligned position while avoiding serious complications. Although the goal is constant from patient to patient, the differences of injury to the pelvis and acetabulum are wide-ranging [14]. It is often challenging to assess the degree of mechanical instability and develop an individualized treatment plan due to variations and patient-specific factors. The application of 3D printing technology can help orthopedic surgeons directly reconstruct the patient’s pelvic or acetabular structure, which assists in the selection of tools and procedures.

The application of 3D printing for pelvic or acetabular fracture surgical planning is becoming a standard practice in hospitals worldwide [15]. The 3D printing models allow surgeons to practice a range of techniques before a procedure. Patients with anterior pelvic ring fracture treated with the virtual simulation and 3D printing-assisted technique have significantly shorter internal fixation times, shorter surgery duration, and less blood loss [16]. The use of 3D printing in preoperative planning for orthopedic trauma surgery suggests that 3D printing reduces operative time, intraoperative blood loss, and the use of fluoroscopy [17]. This study aimed to determine whether applications of 3D printing in traumatic pelvic or acetabular fracture surgery can help shorten the length of hospital stay (LHS) in the adult male population.

## 2. Materials and Methods

### 2.1. Study Design and Participant Selection

This retrospective study enrolled 18–60-year-old male participants with traumatic pelvic or acetabular fractures who visited the emergency department and later received open reduction and internal fixation (ORIF) at the Tri-Service General Hospital in Taiwan from August 2009 to December 2021. The exclusion criteria were (1) patients who died, (2) patients with pathological fracture, (3) patients who received only minimally invasive screw fixation surgery for pelvic or acetabular fracture, and (4) patients who were operated on due to other non-traumatic reasons. All study participants gave written informed consent, and the study followed the protocol approved by the Institutional Review Board of the Tri-Service General Hospital (Approval no.: 2-106-05-092).

### 2.2. Surgical Treatment of Pelvic or Acetabular Fracture

All surgical treatment of pelvic or acetabular fractures was performed by a single experienced trauma orthopedist. The patients received general anesthesia under a relatively stable condition by experienced anesthetists. Depending on the pelvic or acetabular fracture pattern, the ilioinguinal approach, modified Stoppa approach, or Kocher–Langenbeck approach was used by the orthopedist. With the 3D-assisted technique, DICOM (Digital Imaging and Communication in Medicine) computed tomography (CT) scan images were used to process the 3D model images using medical imaging processing software (MIMICS, version 19, Leuven, Belgium). Fused deposition modeling apparatus was used to create patient-specific 1:1 3D printing models (UP BOX+, Tiertime, Beijing, China; Mass Portal XD 40, Mass Portal, Latvia; UP600, Tiertime, Beijing, China; or UP300, Tiertime, Beijing, China). The reconstruction plates were contoured preoperatively. In the surgeries without the 3D-assisted technique, the fixation implants were decided and adjusted intraoperatively. Otherwise, the procedures were performed by the same orthopedist, who specialized in pelvic and acetabular fracture, and the treatment goal was to achieve anatomical reduction and stable fixation [16].

### 2.3. Severity of Trauma

To assess the severity of trauma, we applied the ISS [18]. The ISS was developed from the Abbreviated Injury Scale (AIS) to evaluate the severity and prognosis of trauma [19]. It is an anatomical scoring system in which the body is divided into six parts: the head and neck, face, chest, abdomen, extremities, and body surface. Only the highest AIS score is selected for each part, and then the squares of the AIS scores of the three most injured parts of the body are added together to obtain the total score of the ISS: ISS = (AIS1)^2^ + (AIS2)^2^ + (AIS3)^2^. The total score of the ISS ranges from 0–75 points. An ISS > 15 points is considered major trauma.

### 2.4. Covariates

All the data were obtained from the medical records. Body mass index (BMI) was calculated as weight (in kilograms) divided by the square of height (in meters). Laboratory data were obtained from initial evaluation in the emergency department, including hemoglobin (g/dL), platelet (10^3^/uL), serum glucose (mg/dL), serum creatinine (mg/dL), serum aspartate transaminase (U/L), serum sodium (mmol/L), and serum potassium (mmol/L) levels. The causes of pelvic and acetabular fracture included traffic accidents, falls from height, and other injuries (such as a crushing injury due to a workplace accident or sports). “Duration from admission to pelvic or acetabular fracture surgery” was defined as days from the time the patient first presented to the hospital due to trauma injury until the time the patient received open reduction and internal fixation of these two fractures.

### 2.5. Outcomes

The outcome assessment of this study was focused on the LHS of 3D printing-assisted pelvic or acetabulum fracture surgery. The LHS belonged to the secondary outcome. The primary outcomes of the 3D printing-assisted pelvic or acetabulum fracture surgery (such as internal fixation times, surgery duration, surgical blood loss, and reduction quality) were demonstrated in our previous study [16]. The LHS refers to the number of days the patients spent in hospital. Discharge criteria during the study period were the same between groups: adequate general condition (stable vital signs), clean surgical wound, adequate pain controlled with oral analgesia, could sit in wheelchair, and adequate referral conditions.

### 2.6. Statistical Analysis

We expressed continuous variables with medians and interquartile ranges (IQR, Q1–Q3) and categorical variables with numbers and percentages (%). Descriptive data were compared using chi-square and Mann–Whitney U tests. Pearson correlation was used to analyze the correlation between LHS and continuous parameters. Multivariable regression models were used to analyze the regression coefficients of LHS and the parameters in the comparison of subgroups. The parameters used univariable linear regression to analyze the association with LHS, and the parameters with *p*-values < 0.1 then underwent multivariable linear regression. *p* values < 0.05 were defined as statistically significant. SPSS Statistics for Windows, version 20.0 (SPSS Inc., Chicago, IL, USA), was used for statistical analysis.

## 3. Results

### 3.1. Demographics of the Study Participants

**Figure 1:**
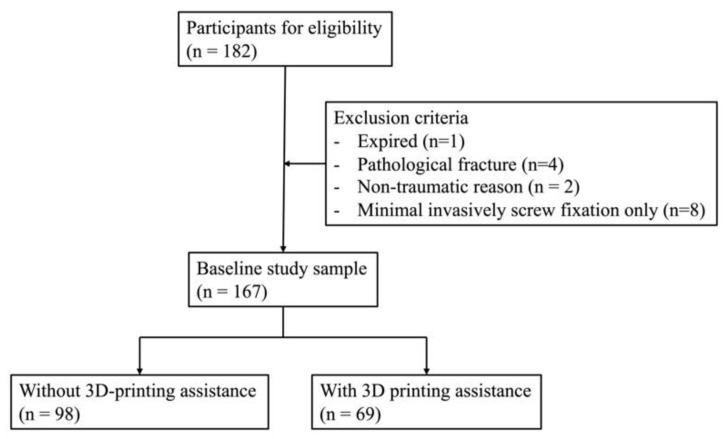
Study flowchart.

There were 182 adult male participants aged 18–60 years who received operative treatment of pelvic or acetabular fracture from August 2009 to December 2021. Patients with pathological fracture (*n* = 4), those who received only minimally invasive screw fixation surgery for pelvic or acetabulum fracture (*n* = 8) due to an old fracture with delayed union (*n* = 2), and those who died (*n* = 1) were excluded. Finally, a total of 167 consecutive patients were included in this study (Figure 1). We divided the participants into patients without 3D printing assistance for pelvic or acetabular fracture surgery (*n* = 98) and patients with 3D printing assistance (*n* = 69). The patients’ demographic characteristics are provided in Table 1. In the group without 3D printing assistance, the median age was 32 (23.75–44) years old. In the group with 3D printing assistance, the median age was 35 (27–48.5) years old. The median ISS of the group without 3D printing assistance was 10 (9–22.75), and that of the group with 3D printing assistance was 9 (9–22). In the groups without and with 3D assistance, the median LHSs were 18 (13–26) and 21 (13–27) days, respectively. The median of the duration from injury to pelvic or acetabular surgery was 4 days in both groups. In the groups without and with 3D assistance, 36.7% and 38.3% of the patients, respectively, received two surgical procedures for pelvic or acetabular fractures. The primary cause of pelvic or acetabular fractures was traffic accidents, especially motorcycle accidents and pedestrians being hit by vehicles (69.5% of total participants), and the second cause was falls from height (26.9% of total participants). Other reasons for pelvic or acetabular fracture included crushing injuries and work accidents. Most of the parameters of the two groups had no significant differences, except platelet levels, but the median platelet levels of both groups were within the normal range.

### 3.2. Measurable Factors and the LHS

Table 2 shows the Pearson correlation between the LHS and the continuous parameters. For all participants, the LHS was positively correlated with ISS, serum glucose, and aspartate transferase (AST) levels and negatively correlated with Hb level. In the group without 3D assistance, the LHS was positively correlated with ISS, glucose level, and AST level and negatively correlated with Hb level. In the group with 3D assistance, LHS was positively correlated with ISS, serum AST level, and the duration from injury to pelvic or acetabular fracture surgery and negatively correlated with Hb level (All *p* values < 0.05).

In Table 3, we divided the participants based on age (under and over 40 years). In the group under 40 years old, LHS was negatively associated with Hb level and positively associated with the number of surgical procedures (*p* value < 0.05) in the multivariable linear regression model. In the group over 40 years old, LHS was positively associated with the ISS, duration from injury to pelvic or acetabular fracture surgery, and the number of surgical procedures in the multivariable linear regression model. Since the ISS was noted to be significantly associated with the LHS regardless of 3D assistance and age, we further analyzed the subgroups of major trauma and non-major trauma, which was defined as an ISS > 15 (Table 4). In the non-major trauma group, LHS was negatively associated with Hb level and positively associated with ISS, duration from injury to pelvic or acetabular fracture surgery, and the number of surgical procedures. In the major trauma group, which had an ISS > 15, LHS was positively associated with the number of surgical procedures in the multivariable regression model. The assistance or lack of 3D printing in pelvic or acetabular operation had no significant association with LHS in either subgroup.

## 4. Discussion

Our study first investigated the effect of 3D printing-assisted pelvic or acetabular surgery in nongeriatric male adults on the LHS. We determined that there is no significant association between the LHS and 3D printing assistance in pelvis or acetabular fracture surgery in 18–60-year-old males. The LHS was associated with several parameters, including ISS, Hb level, serum glucose level, AST level, and the time of surgery. In the younger and major trauma groups, the duration from admission to pelvic or acetabular surgery had no significant association with the LHS. However, in the older and non-major trauma groups, the duration from admission to pelvic or acetabular fracture was significantly associated with LHS. For all subgroups, more surgical procedures led to a longer LHS.

Pelvis and acetabular fractures are rare and challenging fractures in the adult population. The incidence of pelvic ring fractures was found to be 23 per 100,000 persons per year in an Australian study, while the incidence of acetabular fractures was found to be 3 per 100,000 persons per year in a British study [20,21]. In the younger population, the primary cause was high-energy trauma injury. In contrast, in the elderly population, patients may suffer from fractures due to non-high energy injury, such as a fall from standing height. Because of the bone quality difference and the incidence of comorbidities in different age cohorts, we divided the participants into those who were under 40 years old and those who were 40–60 years old. We found that age had no effect on the LHS. Our previous study also found no significant relationship between LHS and age in the elderly population with or without 3D printing assistance [22]. In younger men, the average bone mineral density was within normal range, giving their bones better strength and impact resistance. The pelvis and acetabulum are composed of bony structures and strong ligaments, which is why pelvic and acetabular fractures usually require a high-energy accident, such as high-speed and direct impact traffic accidents, falls from height due to the acceleration of gravity, or crushing injury due to work accidents. The median ISS of patients was also higher in the younger group in this study. The LHS mainly depends on the influence of other organs, and orthopedic surgery is performed until vital organs are stabilized. The conditions of trauma cases are usually complex, and most patients require multiple additional surgeries from the head to the extremities. These may all affect the LHS in the younger cohort.

The two major causes of pelvic and acetabular fractures were traffic accidents and falls from height. About one-fourth of total participants had fall from height injuries in this study, and more than 45% of participants in the older group suffered from falls from height. Different fall distances result in different degrees of trauma. The patients mainly had 3 m, non-intense falls, including falling from a ladder, falling after drunk, or falling from higher distances with deceleration in the older group. Few participants, however, had vital organ trauma. The mortality rate of trauma patients increases with age. Patients may die due to life-threatening trauma in the early stage of trauma care, which reduces the number of patients undergoing pelvic or acetabular fracture surgery. The LHS may also be affected by nosocomial complications, including pneumonia, urinary tract infection, and poor wound healing. One trauma complication study enrolled not only patients who underwent surgery, but also those who underwent non-surgical treatment. Most trauma surgeries are also emergencies. Furthermore, the complications of trauma are different from those of elective surgery [23]. Most patients have healthy or sub-healthy status before trauma. However, the incidence of comorbidities increases with age, which may also affect LHS [24]. These reasons can explain our findings of creatinine and potassium having a significant correlation with the LHS in the older group in the univariable regression model, but not in the multivariable model.

Our research showed that major trauma patients usually have more than two body regions with an AIS score >3 points, indicating relative severity on the six-point ordinal scale, or one item with a score >4 points. However, there was not enough evidence to indicate which part of the injury can be primarily used to predict the LHS. Falls from height and traffic accidents are the most common causes of major trauma. The severity of falls from height is affected by the falling distance, position of landing, whether there is deceleration, and the texture of the impact surface. Falls from height are a common cause of blunt trauma. Age, fall height, fall location, head injury, linear skull fracture, subarachnoid hemorrhage, cervical spine fracture, thoracic spine fracture, and injury score from a different system have a significant effect on mortality [25]. The most common injuries were fractures of the thoracic and lumbar spine, and the incidence of thoracic and pelvic injuries (30.0%) increased after falls from more than 7 m [26]. Traffic accidents are responsible for millions of deaths and injuries yearly. Higher-impact velocities and deformation of merged vehicles resulted in more severe trauma, including that to the head, torso, and extremities. The use of helmets reduced head and neck trauma and overall mortality, but had no protective effect on trunk and extremity trauma [27].

In addition to ISS, we found several statistically significant parameters, including Hb level and whether 3D assistive technology was used. In our study, the lower the Hb level, the longer the hospital stay. Possible reasons for this include lower Hb levels indicating more severe trauma. In our study, the correlation coefficient between ISS and Hb level was −0.365 (*p* value < 0.001). In trauma patients, pelvic and femoral fractures are a common cause of major bleeding, increasing life-threatening hemodynamic instability. Our initial data were collected from patients’ blood draws from their first visit to the emergency department. It was found that during the early trauma care, the patients’ Hb levels could decrease significantly, which may be due to continuous bleeding at the trauma site (trauma in blood-rich organs such as the chest, long bones, liver, and spleen). Hemoconcentration is also often present due to the massive amount of bleeding. The initial Hb blood draw in the emergency department is an important indicator for predicting the severity of trauma. Serum AST level was also found to be significantly correlated with the LHS. Elevated AST levels indicate the possible injury of liver or muscle. Among pelvic and acetabular fracture patients, injury of the abdominal cavity is common. Liver laceration is one of the common complications of traffic accidents and falls from height. Severe trauma usually causes multiple contusions and fractures among the trunk and extremities. High AST levels are associated with severe muscle contusion or possible rhabdomyolysis [28]. Pelvic and acetabular injuries from high-energy trauma frequently cause concomitant internal injuries. Some of these injuries also require emergency surgical intervention. For example, hemorrhagic head injury requires brain surgery, and patients with severe hepatic or splenic injury with a hemodynamic unstable condition require emergency abdominal surgical explorations. This is a possible reason for the positive association between the number of surgical procedures and the LHS in all participants, regardless of the subgroup. The initial evaluations of pelvic or acetabular fracture patients, including Hb level, serum AST level, and ISS, are important indicators for predicting the LHS.

Various 3D application technologies in fracture research have shown that 3D printing has many benefits, including shortening the operation time, improving anatomical reduction, and benefitting the postoperative rehabilitation of patients. In our study, it was found that the LHS could not be shortened with 3D assistance in pelvic or acetabulum fracture patients. Our past research found that in the elderly population, the presence or absence of 3D usage did not shorten the LHS [22]. This study discovered that, in the group with 3D assistance, LHS was significantly associated with the duration from admission to pelvic or acetabular surgery. However, assistance via 3D printing for pelvic or acetabular fracture was not associated with the LHS. Clinically, the expected advantages of 3D assistive technology are different in traumatic patients for reasons other than surgical treatment. In the younger age group, patients with pelvic and acetabular injuries often come to the emergency department with multiple traumas. The goal is to save their life, not obtain functionality. Such patients often have delayed or even no surgery due to other critical lesions. Our study indicated that in non-traumatic patients, a longer duration from injury to pelvic or acetabular fracture may increase the LHS. Generally, nongeriatric adults have healthy or sub-healthy status before trauma injury. Since the non-traumatic group had fewer concomitant injuries that required surgical intervention, the patients could receive pelvic or acetabular surgery earlier, which shortened their LHS. In the age subgroup analysis, younger patients had higher ISSs. The duration from injury to pelvic or acetabular fracture surgery had no effect on the LHS. These patients usually required other surgical interventions for other concomitant injuries prior to orthopedic surgery. They therefore took more time to recover from life-threatening conditions.

There are several limitations to this study. First, this study is a retrospective study. Since pelvic and acetabular fractures are rare, the sample size was small. We did not perform sample size calculations. The variants did not have normal distribution, which may have affected the statistical finding of our results. Second, confounding factors related to patients and interventions were present. Third, this was not a randomized control trial (RCT) study. Complex pelvic fractures are usually diagnosed before surgery, which may have caused bias in patient selection for 3D-assisted pelvic or acetabular fracture surgery. Fourth, although orthopedic surgery was performed by a single physician with rich experience in pelvic surgery, the overall care of trauma patients required the intervention of a multi-specialty team. There are many proven ways to care for patients, but the experience and attitudes of different specialists in caring for patients could have been assessed differently, which may also have affected our experimental results. Fifth, the duration of our study was 12 years, so the initial 3D printing technology, preoperative evaluation, and preoperative preparation technology could not be programmed efficiently. This may have led to the assistance of 3D printing technology prolonging the duration from admission to operation initially. The application of 3D printing technology in orthopedic surgery is now mature, and rebuilt 3D printed models can be completed within 24 h. Given the current technological sophistication, more rigorous study designs, single-team care, and larger-scale prospective studies should be considered in the future for the treatment of pelvic and acetabular fractures.

## 5. Conclusions

Our study revealed statistically significant positive correlation between LHS and ISS, regardless of 3D printing assistance. In the younger and non-major trauma groups, the lower the initial Hb level, the longer the LHS. Among different age groups, the LHS was positively associated with the number of surgeries. In the 40–60-year-old and non-major trauma patients, the duration from admission to pelvic or acetabular fracture surgery was significantly associated with LHS. This study shows that 3D printing-assisted technology for pelvic or acetabular fracture surgery for traumatic nongeriatric male adults does not influence the LHS. More importantly, the evaluation of the initial presentation of trauma patients in the hospital, including the severity of trauma, initial blood test, and the number of operations, are the main predictors of the LHS.

## Figures and Tables

**Table 1:** Demographics.

	Without 3D (*n* = 98)	With 3D (*n* = 69)	*p* Value *
**Continuous Variables, Median (IQR)**			
Age, year	32 (23.75–44)	35 (27–48.5)	0.163
BMI, kg/m^2^	25.06 (22.32–26.87)	25.43 (23.21–27.04)	0.288
Hb, g/dL	13.90 (12.50–14.93)	13.7 (12.3–14.8)	0.502
Platelet, 10^3^/uL	209.00 (167.50–250.25)	251.0 (197.0–301.0)	0.004
Glucose, mg/dL	124 (107–151)	131 (112–162.5)	0.230
Creatinine, mg/dL	0.95 (0.8–1.1)	0.9 (0.8–1.0)	0.732
AST, U/L	39 (27.75–78)	42.0 (31.0–65.5)	0.750
Sodium, mmol/L	138 (136–139)	138 (137–140)	0.157
Potassium, mmol/L	3.7 (3.475–4.0)	3.8 (3.5–4.0)	0.496
ISS	10 (9–22.75)	9 (9–22)	0.428
Length of hospital stay, days	18 (13–26)	21 (13–27)	0.059
Duration from injury to pelvic or acetabular surgery	4 (2–7)	4 (3–9)	0.095
**Category variables, *n* (%)**			
Number of surgical procedures			0.260
One	34 (34.7%)	16 (29.9%)	
Two	36 (36.7%)	28 (38.3 %)	
More than 3	28 (28.6%)	25 (31.7%)	
Mechanism			0.600
Traffic accident	71 (72.4%)	45 (65.2%)	
Falling from height	24 (24.5%)	21 (30.4%)	
Other	3 (3.1%)	3 (4.3%)	

* Chi-square test for categorical variables and Mann-Whitney U Test for continuous variables. Abbreviations: Hb, hemoglobin; AST, aspartate aminotransferase; BMI, body mass index.

**Table 2:** Correlation between LHS and parameters.

	Age	BMI	ISS	Hb	plt	GLU(ER)	Creatinine	AST	Na	K	Duration from Injury to Pelvic or Acetabulur Surgery
Pearson correlation	−0.072	−0.029	0.497	−0.350	0.069	0.153	0.111	0.319	0.023	0.066	0.137
*p* value	0.355	0.736	0.000	0.000	0.376	0.050	0.152	<0.001	0.767	0.399	0.079
**Without 3D**											
Pearson correlation	−0.054	−0.093	0.487	−0.393	0.001	0.273	0.089	0.287	−0.033	0.042	0.128
*p* value	0.598	0.401	<0.001	<0.001	0.990	0.007	0.384	0.004	0.747	0.678	0.212
**With 3D**											
Pearson correlation	−0.122	0.054	0.540	−0.278	0.147	0.014	0.164	0.386	0.101	0.116	0.520
*p* value	0.317	0.706	<0.001	0.021	0.228	0.908	0.177	0.001	0.414	0.344	<0.001

**Table 3:** Regression coefficients of length of hospital stay on the measurable factors in participants with different age subgroups.

	Univariable ^1^		Multivariable ^2^	
	β (SE)	*p* Value	β (SE)	*p* Value
**Age 18–40 (*n* = 105)**				
Age	0.039 (0.241)	0.691		
BMI	0.034 (0.349)	0.825		
Hb	−0.432 (0.595)	<0.001	−0.201 (0.489)	0.013
Platelet	0.025 (0.016)	0.801		
Glucose	0.267 (0.038)	0.006	0.139 (0.051)	0.096
Creatinine	−0.004 (6.286)	0.965		
AST	0.285 (0.008)	0.003	0.099 (0.008)	0.267
Na	0.051 (0.485)	0.610		
K	−0.064 (2.917)	0.516		
ISS	0.464 (0.101)	<0.001	0.091 (0.094)	0.292
With 3D	0.126 (2.879)	0.202		
Duration from injury to pelvic or acetabular surgery	0.101 (0.038)	0.310		
Number of surgical procedures	0.616 (1.450)	<0.001	0.500 (1.364)	<0.001
**Age 40–60 (*n* = 62)**				
Age	−0.235 (0.445)	0.117		
BMI	−0.084 (0.522)	0.588		
Hb	−0.180 (0.851)	0.161		
Platelet	0.151 (0.017)	0.241		
Glucose	0.067 (0.030)	0.609		
Creatinine	0.310 (5.377)	0.014	−0.189 (4.281)	0.059
AST	0.378 (0.011)	0.002	−0.127 (0.009)	0.188
Na	−0.024 (0.675)	0.853		
K	0.269 (3.368)	0.035	0.018 (2.298)	0.836
ISS	0.577 (0.148)	<0.001	0.449 (0.140)	<0.001
With 3D	0.024 (3.398)	0.852		
Duration from injury to pelvic or acetabular surgery	0.627 (0.190)	<0.001	0.359 (0.238)	0.007
Number of surgical procedures	0.606 (1.765)	<0.001	0.516 (1.447)	<0.001

^1^: univariable linear regression, ^2^: multivariable linear regression, Abbreviation: Hb, hemoglobin; AST, aspartate aminotransferase.

**Table 4:** Regression coefficients of Length of hospital stay on the measurable factors in participants with major trauma (ISS > 15).

	Univariable ^1^		Multivariable ^2^	
	β (SE)	*p* Value	β (SE)	*p* Value
**Non-major trauma (*n* = 107)**				
Age	0.021 (0.078)	0.829		
BMI	−0.040 (0.290)	0.718		
Hb	−0.401 (0.537)	<0.001	−0.212 (0.391)	0.002
Platelet	0.237 (0.011)	0.014	0.099 (0.008)	0.128
Glucose	0.167 (0.023)	0.086	0.010 (0.015)	0.875
Creatinine	0.041 (5.199)	0.673		
AST	0.055 (0.037)	0.573		
Na	0.042 (0.433)	0.669		
K	−0.164 (2.539)	0.092	−0.061 (1.650)	0.340
ISS	0.185 (0.482)	0.057	0.150 (0.319)	0.019
With 3D	0.210 (1.984)	0.030	−0.003 (1.340)	0.965
Duration from injury to pelvic or acetabular surgery	0.528 (0.236)	<0.001	0.291 (0.196)	<0.001
Number of surgical procedures	0.678 (1.002)	<0.001	0.492 (0.980)	<0.001
**Major trauma (*n* = 60)**				
Age	−0.057 (0.192)	0.666		
BMI	0.112 (0.515)	0.439		
Hb	−0.132 (0.884)	0.298		
Platelet	−0.059 (0.020)	0.652		
Glucose	0.053 (0.045)	0.691		
Creatinine	0.084 (6.077)	0.525		
AST	0.276 (0.008)	0.034	0.217 (0.008)	0.071
Na	−0.079 (0.637)	0.553		
K	0.234 (3.347)	0.075	0.132 (2.990)	0.257
ISS	0.384 (0.196)	0.002	0.072 (0.189)	0.567
With 3D	0.056 (4.415)	0.672		
Duration from injury to pelvic or acetabular surgery	0.053 (0.044)	0.689		
Number of surgical procedures	0.496 (2.264)	<0.001	0.439 (2.097)	<0.001

^1^ univariable linear regression; ^2^ multivariable linear regression. Abbreviations: Hb, hemoglobin; AST, aspartate aminotransferase.

## Data Availability

Data are contained within the article.
